# Virtual reality cue exposure therapy for tobacco relapse prevention: a comparative study with standard intervention

**DOI:** 10.1017/S0033291722002070

**Published:** 2023-08

**Authors:** Eric Malbos, Baptiste Borwell, Mélodie Einig-Iscain, Théo Korchia, Robin Cantalupi, Laurent Boyer, Christophe Lancon

**Affiliations:** 1 Department of Adult Psychiatry, Conception University Hospital, Marseille, France; 2 Equipe Imothep, Institut Fresnel, UMR 7249, Aix-Marseille Université, CNRS, Ecole Centrale Marseille, Marseille, France; 3 Cognitive Psychology Lab, UMR 7290, Aix-Marseille University, Marseille, France; 4 CEReSS, EA 3279, Center, La Timone Faculty of Medicine, Aix-Marseille Université, Marseille, France

**Keywords:** Cue exposure therapy, game level editor, tobacco relapse prevention, virtual environments, virtual reality

## Abstract

**Background:**

Successful interventions have been developed for smoking cessation although the success of smoking relapse prevention protocols has been limited. Cognitive behavioural therapy (CBT) in particular has been hampered by a high relapse rate. Because relapse can be due to conditions associated with tobacco consumption (such as drinking in bars with friends), virtual reality cue exposure therapy (VRCE) can be a potential tool to generate 3D interactive environments that simulate risk situations for relapse prevention procedures.

**Methods:**

To assess the effectiveness of VRCE with CBT, a comparative trial involving 100 smoking abstinent participants was designed with all required virtual environments (VE) created with an inexpensive graphic engine/game level editor.

**Results:**

Outcome measures confirmed the immersive and craving eliciting effect of these VEs. Results demonstrated that more participants in the VRCE group did not experience smoking relapse and that VRCE is at least as efficacious as traditional CBT in terms of craving reduction and decrease in nicotine dependence. Dropout and relapse rate in the VRCE group was noticeably lower than the CBT group. Aside from mood scores, no significant differences were found regarding the other scales.

**Conclusion:**

**The present clinical trial provides evidence that VRCE was effective in preventing smoking relapse.** Improvement in technology and methodology for future research and applications is delineated.

## Introduction

Drug craving is defined as a strong *impetus* or desire to use substances and is generally viewed as a central feature of addiction (Sayette et al., 2000; Tiffany, 1990). It has been associated with drug consumption maintenance (Bagot, Heishman, & Moolchan, 2007; Brandon, Piasecki, Quinn, & Baker, 1995; Shiffman, Engberg, & Paty, 1997) and has been described as a barrier for individuals trying to quit (Baker, Mermelstein, & Collins, 2011). In particular, nicotine dependence is the leading cause of preventable morbidity, mortality and health expenses in developed countries (World Health Organisation, 2009). The World Health Organization, in its WHO Report on the Global Tobacco Epidemic, reported that if current tobacco use persists, it will cause the death of more than 8 million people worldwide every year by the year 2030 (World Health Organisation, 2009). This dependence involves compulsive use despite the awareness of adverse consequences and repeated cycles of abstinence and relapse. The high rates of relapse after smoking cessation programmes have ranged between 40% and 70%, suggesting the need to incorporate more effective strategies for relapse prevention into such programmes (Hatsukami, Stead, & Gupta, 2008). To better understand the underlying mechanisms of relapse, a cognitive behavioural model was developed by Marlatt and Gordon, in which relapse to drug use was usually associated with high-risk situations characterized by the presence of drug-related stimuli (Marlatt & Gordon, 1985). In addition, several studies have reported that individuals with substance use disorders have physiological and subjective reactions to the presentation of drug-related stimuli, a phenomenon known as cue reactivity (Carter & Tiffany, 1999). Recent studies have also reported that cue-induced craving does not decrease over an extended period of abstinence and might actually increase with a longer duration of abstinence (Bedi et al., 2011). Nowadays, several tobacco quitting methods exist such as psychotherapy, nicotine replacement therapy (NRT) and electronic cigarettes. Still, a significant challenge lies in the prevention of relapse (Hatsukami et al., 2008). Indeed, unlike what can be observed with other substance abuse, where risk situations are highly specific and can be easily avoided, cigarette smoking is usually related to daily situations that either cannot or should not be avoided (i.e. workplace and coffee break with colleagues, friends smoking during social events or in public places). In this particular context, two techniques have emerged as potential smoking relapse prevention interventions: cognitive behavioural therapy (CBT) and cue exposure therapy (CET). CBT aims to explain and treat the individual's cognitive dysfunctions and habits (i.e. dysfunctional beliefs and automatic thoughts related to smoking). This method can increase the individual's motivation and can play an important role in relapse prevention through analysis and understanding of this phenomenon (Guichenez et al., 2007). However, a meta-analysis reported insufficient evidence to support the use of CBT to prevent relapse, and advised the evaluation of alternatives in attempts to teach coping skills in risk situations (Hajek et al., 2013). On the other hand, CET is a behavioural approach to drug dependence based on the classical conditioning model (Drummond, Tiffany, Glautier, & Remington, 1995). This treatment involves extinction procedures wherein addict patients are repeatedly exposed to drug-related cues with the aim of decreased reactivity (Carter & Tiffany, 1999). CET can be added to CBT and applied through several modalities of exposure including videos, mental imagery procedures and *in vivo* presentations of cues. However, these situations are difficult to reconstruct effectively in passive video or images as well as in the artificial context of a hospital or an office, thus limiting the efficacy of CET. Because of the complexity of nicotine cue reactivity involving proximal (lit cigarette, ashtray, lighter), contextual (physical situations such as a party or a bar) and complex cues (a combination of contextual and proximal cues, such as situations involving social interactions where people are smoking or offered cigarettes), more ecological environments should be proposed than those used in traditional CET (Traylor, Parrish, Copp, & Bordnick, 2011).

These observations address *de facto* the need to implement new exposure strategies to help abstinent smokers cope with smoking-related situations in an active way. Consequently, virtual reality, an immersive media allowing subjects to be exposed and interact in computer-generated environments in real time has been considered an option. When virtual reality exposure is associated with drug-related stimuli, this method is entitled virtual reality cue exposure (VRCE) and has been recently under examination as a possible alternative instrument to traditional CBT (García-Rodríguez, Valverde, Maldonado, & García, 2009). In the specific field of nicotine dependence treatment, past studies have listed VRCE's possible advantages such as the realistic simulation of situations related to drug use, simultaneous presentation of proximal and distal tobacco cues and *ad libitum* re-experiencing (Baumann & Sayette, 2006). Thus, the use of VR within smoking cessation programmes could be a relevant approach (Lee, Lim, Graham, Kim, & Wiederhold, 2004). Even though previous researches have studied VRCE and demonstrated that artificial 3D situations can induce tobacco craving with success or lead to decrease in nicotine addiction (Girard, Turcotte, Bouchard, & Girard, 2009; Pericot-Valverde, Secades-Villa, Gutiérez_Maldonado, & Garcia-Rodriguez, 2014), the efficacy of VRCE on relapse prevention has yet to be analysed.

Therefore, this study sought to investigate the effect of VRCE on smoking relapse prevention in the context of a comparative study involving VRCE and traditional CBT/CET. The objectives of this trial are threefold. The main goal was to evaluate the effectiveness of VRCE compared to traditional CBT/CET in the prevention of smoking relapse and the effect on dependence and craving. Secondly, to measure the impact of VRCE on other psychological features including depression, anxiety, self-esteem and quality of life. Thirdly, this assay aims to ensure that the six virtual environments (VEs) constructed for the experiment produced craving and presence with limited cybersickness. Indeed, a key to successful immersion and therapy is this sense of presence, which has been frequently defined as a feeling of transportation: a sensation of being present in a virtual world (Lombard & Ditton, 1997).

The overall efficacy and immersive properties of the prevention protocol and its VEs were assessed with self-report questionnaires as well as and physiological measures.

## Methodology

All participants gave written informed consent to their participation in this study in accordance with the Declaration of Helsinki, and the study was approved by the Ethics Institutional Review Board CPP Sud Méditerranée. The present trial was registered on ClinicalTrials.gov (Identifier: NCT02205060) and a comprehensive report on the methodology was previously published before the completion of the recruitment process (Giovancarli et al., 2016).

### Sample

One hundred participants (71 women, 29 men) meeting DSM-5 criteria for recent chronic smoking were recruited for the clinical trial through local media and onsite consultations. The inclusion criteria were as follows: male or female, aged of 18 years or more with a past diagnosis of chronic smoking as defined by the DSM-5 and with the presence of at least three of the 11 DSM-5 criteria for nicotine dependence (American Psychiatric Association, 2013). Additionally, the participants had to report smoking abstinence for at least 1 week (defined by the total absence of tobacco consumption reported by the individual) and their current abstinence was verified by measuring the carbon monoxide (CO) exhaled at pretest. The accepted threshold for abstinence followed past recommendations for optimal sensitivity and sensibility: a CO exhaled levels of less than 4 parts per million (ppm) (Javors, Hatch, & Lamb, 2005). In order to avoid possible contamination by other therapeutic means, the main exclusion criteria were the simultaneous use of any concurrent method of tobacco abstinence (i.e. NRT, smoking cessation drugs such as bupropion or varenicline, electronic cigarettes, hypnosis). Other exclusion criteria were as follows: pregnancy or breastfeeding; unstable physical or psychiatric disease and contraindications to virtual reality therapy such as photosensitive epilepsy. Sociodemographic characteristics are listed in Table 1.

**Table 1.** Social and demographics characteristics of the sample

Characteristics	Total *n* = 100 (SD)	CBT *n* = 50	VRCE *n* = 50
Age	47.65 (13.31)	47.32 (14.55)	47.98 (12.08)
Sex			
Female	71	35	36
Male	29	15	14
Marital status			
Single	28	13	15
Married/*de facto*	55	29	26
Divorced	16	7	9
Widower	1	1	0
Education			
Secondary	17	10	7
Tertiary	83	40	43
Videogames experience			
Playing every day	9	5	4
Playing more than once a week	9	5	4
Playing less than once a week	10	5	5
Stopped playing	32	17	15
Never played	40	18	22

s.d., standard deviation.

### Assessments

The primary measure was the proportion of individuals with maintenance of their tobacco abstinence at the completion of the protocol (Lancaster, Stead, & Cahill, 2008; Mottillo et al., 2009). Abstinence is defined by the total absence of tobacco consumption as assessed during a post-treatment inquiry and the systematic use of a CO breath monitor that measures the CO levels exhaled for the sake of a more objective evaluation. Should the results be greater than or equal to the cut-off point of 4 ppm, the participant was considered as having a relapse (Javors et al., 2005; Underner & Peiffer, 2010). Other addiction and global psychological well-being psychometric measures include:

*Cigarette Dependence Scale* (CDS-12) (Etter, Houezec, Huguelet, & Etter, 2009), a 12-item self-report instrument which measures the severity of cigarette dependence with scores ranging from 12 (no dependence) to 60 (high dependence). This scale has high test-retest reliability (⩾0.83) and high internal consistency (Cronbach's *α* ⩾0.84).

*French Tobacco Craving Questionnaire* (FTCQ-12) (Berlin, Singleton, & Heishman, 2010), a 12-item self-report instrument with scores ranging from 12 (no craving) to 84 (high craving). The internal consistency *α* coefficients were 0.83, 0.79, 0.69 and 0.66 for the different factors (emotionality, expectancy, compulsivity, and purposefulness). In session craving itself is also evaluated using an analogical craving scale ranging from 0 (no urge) to 100 (extreme urge) that measures the perceived level of craving at a given time during the exposure to virtual or imaginary smoking-related cues (Sayette & Hufford, 1994) during each session.

*State-Trait Anxiety Inventory* (STAI) Y-A (state) (Gauthier & Bouchard, 1993). The STAI is a 20-item self-report instrument with scores ranging from 20 (absence of anxiety) to 80 (high anxiety). The STAI is among the most widely researched and widely used measurements of general anxiety, with satisfactory internal consistency *α* coefficients (⩾0.7).

*Beck Depression Inventory* (BDI) (Collet & Cottraux, 1986; Steer, Beck, Riskind, & Brown, 1986). The BDI is a 13-item self-report instrument. A total score between 4 and 7 shows a mild state of depression, between 8 and 15 an average to moderate state of depression and 16 or higher a severe state of depression. This scale has satisfactory psychometric properties. A meta-analysis of the BDI's internal consistency estimates yielded a mean *α* coefficient of 0.81. The concurrent validity of the BDI with respect to clinical ratings and the Hamilton Psychiatric Rating Scale for Depression was also high. The BDI also distinguishes subtypes of depression and differentiates depression from anxiety (Beck, Steer, & Carbin, 1988).

*SF-12* (Ware, Kosinski, & Turner-Bowker, 2002). Quality of life was assessed with this 12-item scale assessing physical function, physical pain, general health, vitality (energy and tiredness), social functioning and well-being as well as limitations due to physical and mental health. Two composite scores are obtained with this self-report instrument: a Physical Component Score (PCS) and a Mental Component Score (MCS). A high score indicates a high-level quality of life. Several studies have reported that the SF-12 is able to produce the two summary scales originally developed from the SF-36, one of the most widely used quality of life instruments, with considerable accuracy and yet with far less of a respondent burden (Jenkinson & Layte, 1997).

*Rosenberg Self-esteem scale* (*EES*) (Rosenberg, 1965). This is a self-report questionnaire related to self-esteem and consisting of 10 items which produces a total score of 10–40; a high score indicates high self-esteem. This questionnaire is a reliable and valid measurement of global self-worth (Gray-Little, Williams, & Hancock, 1997).

In addition to the instruments measuring the psychological and physiological impact, when using virtual reality, a specific questionnaire related to presence (the Presence Questionnaire) is usually required. The most common definition of presence which pertains to the immersion in VEs is the one related to the concept of transportation to elsewhere: the feeling of ‘being there’ (Lombard & Ditton, 1997) that is the pregnant impression of being existent in a real or artificially created place (Malbos, Rapee, & Kavakli, 2013).

In the framework of experimental conditions where ecologically valid situations are required for the study of behaviour or reactions, presence is of significant importance. Indeed, a key requirement is that a subject will behave in a VE as they would when confronted to similar cues in real life. Similar characteristics are required for the use of virtual reality in clinical interventions.

Side effects of using virtual reality and related apparatus are summarized as cybersickness, a form of motion sickness which induces various symptoms (such as nausea, sweating, dizziness) when the user is immersed in VEs. Following past recommendations(Robillard, Bouchard, & Fournier, 2003), measuring cybersickness through questionnaires such as the Simulation Sickness Questionnaire (SSQ) is especially important since cybersickness may hinder perceived presence and consequently the treatment efficiency.

Presence and cybersickness were registered after each VR exposure session in the VRCE group using the *Presence Questionnaire PQ v3.0* (Witmer & Singer, 1998) and the *SSQ* (Kennedy, Lane, Berbaum, & Lilienthal, 1993). The PQ consists of 32 items rated on a seven-point scale, and factor analysis consists of six factors: involvement, interface quality, adaptation and immersion, consistency with expectation, visual fidelity and auditory fidelity (Witmer & Singer, 1998). The PQ is a self-report instrument that has been validated in many empirical studies (Kennedy et al., 1993; Price & Page, 2007; Witmer, Jerome, & Singer, 2005; Witmer & Singer, 1998) and has also demonstrated a relationship between presence and anxiety (Price & Page, 2007). A high score indicates a satisfactory perception of presence.

The SSQ is a 16-item self-report instrument with scores ranging from 16 (absence of cybersickness) to 48 (high cybersickness). Factor analysis consists of three main factors: oculomotor (i.e. blurred vision), disorientation (i.e. dizziness) and nausea (i.e. vomiting).

Heart rate (HR) and heart rate variability (HRV) were monitored and carried out throughout each session in the VRCE group using a Polar RS800CX™ device, which includes a transmitter and a wrist receiver. This monitor has proved its efficacy in clinical research (Quintana, Heathers, & Kemp, 2012). The HRV indicates the fluctuations in HR around an average HR (Vanderlei, Silva, Pastre, Azevedo, & Godoy, 2008). HRV was assessed by calculating a time domain variable entitled root mean square of successive differences (RMSSD) which is the square root of the mean squared difference of successive RR or NN waves, and by the pNN50 which is the proportion of adjacent R or N waves more than 50 ms (Vanderlei et al., 2008). HR and HRV reflect the autonomic responses involved in emotional arousal, most notably during anxiety or craving where the HR is expected to increase and the HRV to decrease (Bernston & Cacioppo, 2007; Carter & Tiffany, 1999). There is also evidence that HR changes are correlated with presence (Wiederhold, Jang, Kaneda, Cabral, & Lurie, 2003). HR and HRV represent an alternative objective measurement of anxiety response, presence and craving.

### Apparatus and virtual environments

The VR system includes a ruggedized Sensics ZSight HMD (1280 × 2024 stereoscopic OLED screen with 60° field of view), coupled with an embedded 3 degrees of freedom head tracker (angular resolution: 0.05°, latency 8 ms). The head tracker enables the participant to visually explore the environment by updating the 3D scene as a function of head orientation. Otherwise, navigation is triggered by mouse motion. The participant's locomotion direction is defined by his or her head orientation in the VEs. The participants must use a wireless controller with a directional pad for walking or swimming locomotion. The steering wheel exploited for driving VE was a Logitech G29 with vibration and force feedback capabilities. The VEs are generated and run on an ordinary graphics-orientated notebook with a four-core processor, 16Go DDR2 RAM, a graphics card with 3 Go RAM and a 1440 × 900 resolution screen. The required software is Microsoft Windows 10 (64-bit edition), Microsoft DirectX 9.0 or higher and the equipment's drivers.

The main software exploited to create and run the VEs was Sandbox. Sandbox was an inexpensive (30–60 Euro/USD) and commercially available game level editor (GLE) of the video game Crysis 1/2, exploiting the CryEngine graphic engine developed by Crytek GmbH. Prior to its full use for the trial, this GLE was tested and compared to seven other commercially available GLEs by considering several distinct criteria and requirements previously reported (Malbos, Rapee, & Kavakli, 2011). To construct the VEs, the investigator exploited the aforementioned GLE to build six specific cue-graded VEs related to smoking. The VEs were selected to represent common situations of daily life involving high-risk situations in terms of smoking relapse (Garcia-Rodriguez, Ferrer-Garcia, Pericot-Valverde, Gutierrez-Maldonado, & Secades-Villa, 2011; Garcia-Rodriguez, Pericot-Valverde, Gutierrez, Ferrer-Garcia, & Secades-Villa, 2012). These VEs are validated smoking eliciting situations according to empirical works on tobacco consumption (Beck, Wright, Newman, & Liese, 2001; Marlatt & Gordon, 1985), past studies (Girard et al., 2009; Pericot-Valverde et al., 2014) and the criteria of the DSM-5 (American Psychiatric Association, 2013). These six VEs offer distinct craving-inducing scenarios: having a drink with people smoking in a virtual beach bar at sunset; walking with avatars smoking on the terrace of a restaurant; being in a furnished living room or its balcony with a beer, an ashtray and a lighted cigarette; waiting at a bus stop with avatars smoking around; taking a break in a workplace with smoker colleagues and driving a virtual car on a road during a traffic jam.

**Fig. 1.**
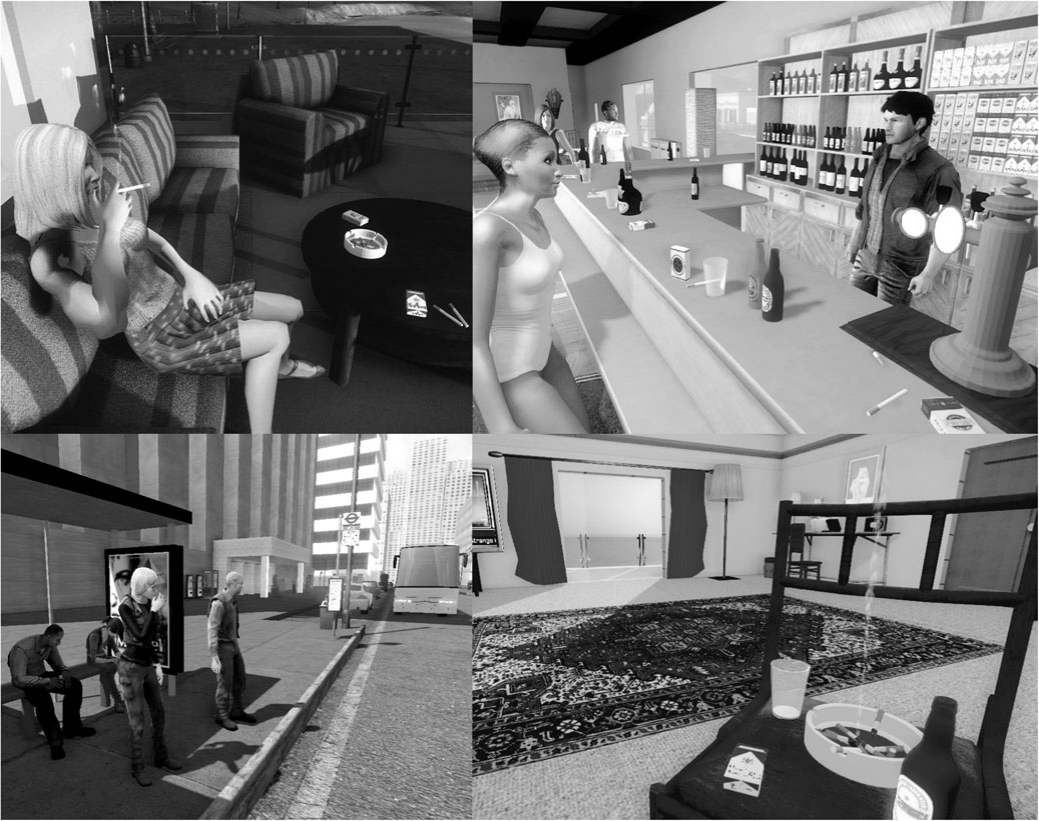
Screenshots of four VEs constructed for the present study. Note the smoking-related cues (cigarettes and packets, ashtray, alcohol) and the avatars' smoking attitudes (beach bar, restaurant, bus stop and interior with balcony).

During exposure, the investigator can trigger specific events within the VE (i.e. avatars talking about smoking or inviting the participants to smoke a cigarette or drink a cup of coffee). These options allow for progressive increases in the intensity of induced craving to modulate the degree of exposure at various times. Dynamic VEs also provide the participant with direct, realistic interactions (such as opening doors, virtual human interactions, grabbing objects and physical or mechanical reactions to the user's presence) (Fig. 1).

### Procedure

Following the intake assessment and the diagnostic interview, the participants were randomly assigned to two therapeutic groups: one group receiving VRCE and one group receiving traditional CBT. The allocation to each group was determined using a stratified random sampling method. The protocol included 8 weekly sessions of 45 min each for both groups. All participants were taught addiction centred psychoeducation (addiction modelization), craving management, relaxation, positive self-statements, assertiveness (how to refuse a cigarette) and cognitive restructuring as outlined by several works of reference (Beck et al., 2001; Marlatt & Gordon, 1985). The first two sessions were entirely dedicated to acquiring these methods while following sessions provided additional instruments before exposure. The only difference between the groups was the smoking related exposure procedure carried out from the 3rd to 8th session. While participants in the CBT group were asked to visualize such conditions, the participants in the VRCE group were immersed in a computerized world using virtual reality equipment. Both methods aim to reduce craving or cue reactivity by habituation or extinction. Moreover, throughout the exposure sessions, the participants were regularly encouraged to review and apply the methods acquired previously. Given individual differences in the relevance of craving-specific stimuli and to determine the order of exposure to virtual or imagined situations, a list was established with each participant independently, from the least craving inducing to the most craving inducing environment. In the VRCE group, VR exposure was presented in gradual context, since smoking-related cues can vary in intensity (i.e. number of avatars smoking around the patient, presence of cigarette boxes spread on tables). Throughout the exposure to VEs, the participants were invited to progress to the next VE when they had reached a level of emotion or craving considered as comfortable in the current environment (between 0 and 20 on the craving scale). Additionally, to optimize habituation or extinction, exposure to a particular event or situation could be repeated at the participant's discretion.

## Results

**Fig. 2.**
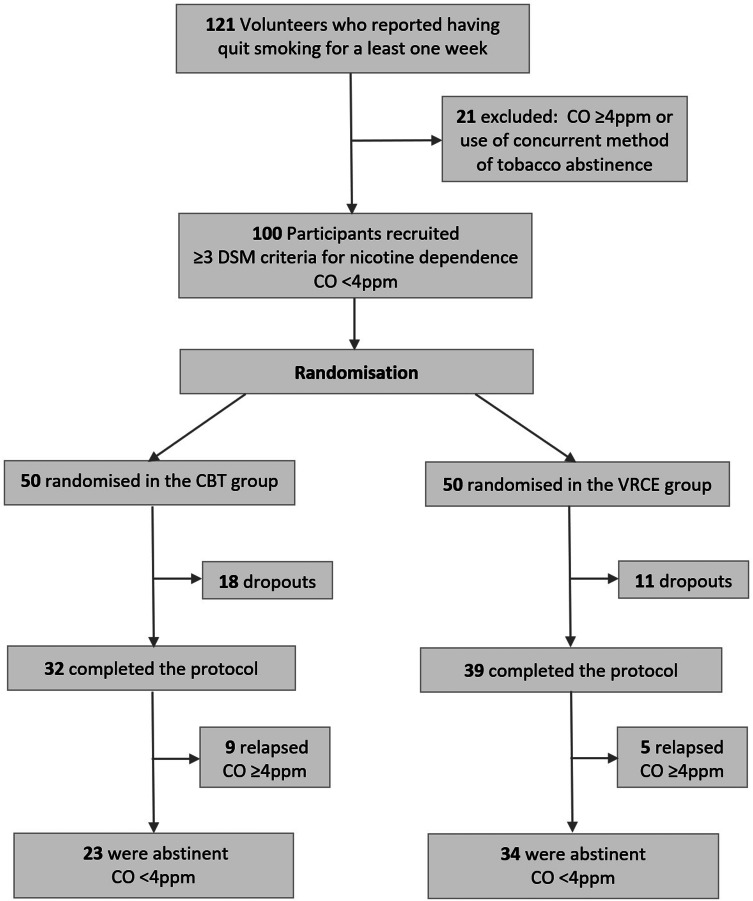
Flowchart of the different stages of inclusion and progression through the protocol.

The progression of participants through the protocol is summarized in the flowchart (Fig. 2). Twenty-nine participants dropped out at different stages of the protocol for different reasons (18 in the CBT group *v.* 11 in the VRCE group).

Regarding the cumulative choice of VEs over the course of the six sessions in the VRCE group, 32.3% of the participants selected the beach bar, 22.3% the workplace with a break, 17.1% the living room or balcony, 11.7% the restaurant, 9.2% the bus stop, 7.1% the traffic jam.

### Effects of virtual reality exposure on craving, presence and cybersickness

**Fig. 3.**
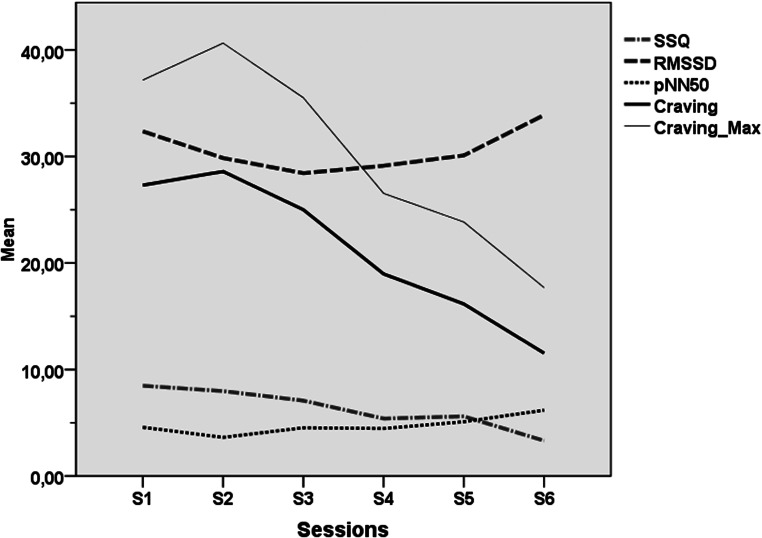
Line representation of mean SSQ, mean and maximum craving, RMSSD and Pnn50 across all exposure sessions in the VRCE group (sessions 3–8). SSQ, Simulation Sickness Questionnaire; RMSSD, root mean square of successive differences (ms); pNN50, proportion of adjacent R waves more than 50 ms (%).

**Fig. 4.**
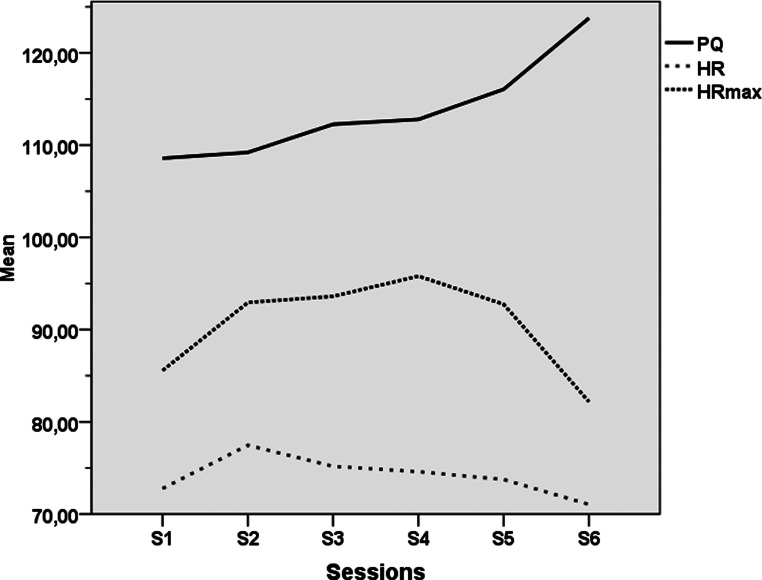
Line representation of mean PQ, HR and HRmax across all exposure sessions in the VRCE group (sessions 3–8). PQ, Presence Questionnaire; HR, mean heart rate per min; HRmax maximum heart rate per min.

In the VRCE group, the VEs were able to produce tobacco craving among the participants with a mean craving level at 21.26/100 (s.d. = 9.97) across all sessions and at 28.59/100 (s.d. = 16.01) at its peak during the fourth session and a maximum craving level 30.23/100 (s.d. = 12.36) at across all sessions and at 40.64/100 (s.d. = 20.84) at its peak during the fourth session (cf. Fig. 3). For the following sessions (S5 to S8), craving scores decreased progressively and significantly (cf. Table 2 and Fig. 3). Regarding presence and possible VR side effects, mean PQ high scores (PQ = 113.77, s.d. = 13.98) and mean SSQ low scores (SSQ = 6.30, s.d. = 5.07) indicated that the participants felt immersed in the VEs without exhibiting strong cybersickness. The PQ raised over time significantly while the SSQ was ebbing away as detailed in Table 2 and represented in Figs 3 and 4.

**Table 2.** Mean, standard deviations of the dependent variables, results of two-way ANOVA between the third session S3 (beginning of exposure session) and eighth session S8 (post-test) (time)

Tests	Group	S3 mean (s.d.)	S8 mean (s.d.)	ANOVA time *F* (1,69)	Eta^2^ time	ANOVA interaction *F* (1,69)	Eta^2^ interaction
Craving S3S8	CBT	22.50 (22.9)	11.40 (14.65)	50.12***	0.42	1.52 ns	0.02
	VRCE	27.30 (17.80)	11.53 (11.87)				
PQ S3S8	VRCE	108.58 (16.80)	123.76 (15.8)	36.94***	0.50		
SSQ S3S8	VRCE	8.47 (7.71)	3.31 (3.32)	23.54***	0.39		
HR S3S8	VRCE	72.78 (8.04)	71.05 (9.09)	1.04 ns	0.03		
HRmax S3S8	VRCE	85.57 (16.57)	82.19 (18.55)	0.65 ns	0.02		
RMSSD S3S8	VRCE	32.36 (6.54)	33.89 (5.69)	1.25 ns	0.03		
pNN50 S3S8	VRCE	4.57 (1.88)	6.18 (2.68)	7.60**	0.17		

ANOVA, analysis of variance; VRCE, virtual reality cue exposure; PQ, Presence Questionnaire; SSQ, Simulation Sickness Questionnaire; Craving, mean craving level; HR; HRmax, maximum heart rate per min; RMSSD, root mean square of successive differences (ms); pNN50, proportion of adjacent R waves more than 50 ms (%).

***p < 0.001; **p < 0.025; *p < 0.05; ns: non-significant.

### Effects of treatment on symptoms and physiology

**Fig. 5.**
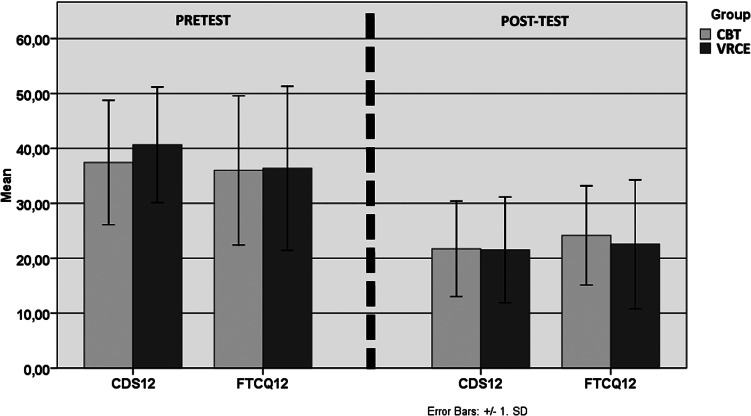
Representation of mean and standard deviations of the dependent variables between pretest and post-test. VRCE, virtual reality cue exposure; CBT, cognitive behavioural therapy; CDS12, Cigarette Dependence Scale; FTCQ12: French tobacco Craving Questionnaire.

Questionnaire scores and physiological measures are detailed in Tables 2 and 3 and represented in Figs 3–5. A two-way mixed analysis of variance (ANOVA) was used to compare the two groups over time (pretest *v.* post-test) and interaction (VRCE *v.* CBT). There is evidence of significant main time effects for all subjective measures related to smoking dependence and tobacco craving namely the CDS-12, FTCQ-12 and in session mean craving. Significant time effects were also found for the BDI but not for the two factors of the SF-12 nor the EES. Regarding group comparison, examination of group by time interactions indicated no significant interactions for any variable. However, according to the record of CO exhaled upon finishing the protocol with a cut-up value at 4 ppm, five participants (7.1%) of the VRCE group and nine (12.8%) in the CBT group were in relapse (cf. Table 4).

**Table 3.** Mean, standard deviations of the dependent variables, results of two-way ANOVA between pre- and post-test period (time), between the third session (beginning of exposure session) and eighth session (post-test), and ANOVA for time × group comparison (interaction)

Tests	Group	Pre-test mean (s.d.)	Post-test mean (s.d.)	ANOVA time *F* (1,69)	Eta^2^ time	ANOVA interaction *F* (1,69)	Eta^2^ interaction
CDS-12	CBT	37.34 (11.33)	21.71 (8.69)	154.79***	0.69	1.48 ns	0.02
	VRCE	40.65 (10.52)	21.52 (9.61)				
FTCQ-10	CBT	36 (13.56)	24.15 (9.03)	81.71***	0.55	0.48 ns	0.01
	VRCE	36.36 (14.93)	22.55 (11.72)				
STAI Y-A	CBT	33.50 (7.87)	34.03 (10)	0.19 ns	0.00	0.73 ns	0.01
	VRCE	32.43 (9.85)	30.74 (11.02)				
BDI	CBT	15.87 (2.47)	15.28 (2.49)	10.41**	0.13	2.04 ns	0.03
	VRCE	17 (3.96)	15.46 (4.02)				
SF12ph	CBT	51.11 (5.40)	50.40 (7.78)	0.44 ns	0.01	2.58 ns	0.04
	VRCE	49.75 (7.98)	51.43 (5.71)				
SF12me	CBT	47.97 (6.47)	49.13 (6.74)	3.02 ns	0.04	0.20 ns	0.00
	VRCE	46.68 (9.74)	48.66 (1)				
EES	CBT	33.46 (5.21)	32.71 (6.67)	0.24 ns	0.00	2.83 ns	0.04
	VRCE	31.51 (5.08)	32.87 (3.53)				

ANOVA, analysis of variance; VRCE, virtual reality cue exposure; CBT, cognitive behavioural therapy; CDS, Cigarette Dependence Scale; FTCQ, French tobacco Craving Questionnaire; STAI, State-Trait Anxiety Inventory; BDI, Beck Depressive Inventory. SF12ph/me: physical and mental factors of the SF12; EES: Rosenberg Self-esteem Scale.

***p < 0.001; **p < 0.025; *p < 0.05; ns: non- significant.

**Table 4.** Distribution of participants according to CO exhaled at post-test

Post-test CO	<4 ppm	<10 ppm	<20 ppm	>20 ppm
% Participants (n = 71)	80.3%	8.4%	9.8%	1.5%
% Participants VRCE	87.2%	5.2%	5.2%	2.5%
% Participants CBT	71.9%	12.5%	15.6%	0%

CO, exhaled carbon monoxide; ppm, parts per million

With respect to the analysis of cardiophysiological recordings in the VRCE group as reported in Table 2 and Fig. 4, a slight decrease of the average HR and maximum HR between the first and the last VR exposure session can be observed, although it did not reach statistical significance. Concerning the HRV parameters and their elevation between the first and the last VR exposure session, a significant time effect for the pNN50 was found (S3 to S8: *F* = 7.60; *p* < 0.01).

### Correlation

For systematic analysis, correlations were evaluated between demographic values using Pearson calculation. These results demonstrated significant negative correlations between video game experience and age (*r* = −0.246; *p* < 0.04), and between marital status and level of education (*r* = −0.28; *p* < 0.02).

Regarding presence, the influence of gaming experience on immersion was verified: there was minimal non-significant correlation between overall PQ and gaming experience (*r* = −0.12; *p* < 0.48). Interaction between presence and craving was also evaluated: small non-significant positive correlations were found between PQ and overall maximum craving (*r* = 0.21; *p* < 0.21) but not between PQ and overall mean craving (*r* = 0.09; *p* < 0.57). Moreover, to investigate any relationship between perceived presence and treatment efficacy, difference scores were calculated between pre-test and post-test for all dependent variables and correlations were computed between these scores, and mean PQ across all sessions. Large to minimal significant or non-significant positive correlations were found between PQ and pre-post difference for BDI (*r* = 0.61; *p* < 0.019), FTCQ-12 (*r* = 0.51; *p* < 0.38), SF-12 physical (*r* = 0.18; *p* < 0.53), CDS-12 (*r* = 0.13; *p* < 0.83) and STAI-A (*r* = 0.10; *p* < 0.95). Small to medium significant or insignificant negative correlations were found between PQ and pre-post difference for EES (*r* = −0.36; *p* < 0.19) and SF-12 mental (*r* = −0.23; *p* < 0.42).

With respect to the effect of presence on physiological parameters evolution, HR and HRV difference rates were calculated between the third and last sessions, small to medium insignificant positive correlations were noted between PQ and mean HR (*r* = 0.27; *p* < 0.11) and maximum HR (*r* = 0.30; *p* < 0.076). Small to medium insignificant negative correlations were noted between PQ and RMSSD (*r* = −0.21; *p* < 0.22) and pNN50 (*r* = −0.15; *p* < 0.36).

No correlations were computed through the six exposure sessions between PQ and SSQ scores on one hand and the craving/physiological scores on the other hand, as their distinct evolution over time indicated a different linear/non-linear pattern (cf. Figs 3 and 4).

We hypothesized that during initial VR sessions, it was common to observe slightly more discomfort when using the VR apparatus if the participants were not familiar with its use. An increase in HRV as well as some symptoms such as nausea can be both triggered by the parasympathetic system (Lanier, Minsky, Fisher, & Druin, 1989; Riva et al., 2015). Thus, correlations were searched between cybersickness and physiological measures. However, for the first VR session, no correlations were demonstrated between SSQ and RMSSD (*r* = 0.09; *p* < 0.58), pNN50 (*r* = 0.01; *p* > 0.97) and mean HR (*r* = −0.04; *p* < 0.79).

## Discussion

The present clinical trial provides evidence that VRCE was effective in preventing smoking relapse with a greater effect than traditional CBT/CET: more participants in the VRCE group who completed the protocol did not experience smoking relapse as attested by post-test exhaled CO. Additionally, there were fewer dropouts in VRCE group. We posit that the participants in the VRCE group were more motivated considering that several past trials related to anxiety disorders treatment demonstrated a general preference for VR upon other traditional exposure methods [35].

Other outcomes revealed a significant reduction in tobacco craving and dependence in both groups, and aside from smoking-related variables, a small increase in mood was also observed. However, no significant differences were found regarding the other scales, even though an improvement in mental quality of life was observed but was not enough to reach statistical significance. Group comparison between all variables did not lead to any significant interactions, indicating that VRCE is at least as effective as traditional CBT/CET in terms of craving and dependence. When considering cardiophysiological measures in the VRCE group, after an initial increase of cardiac activity, the VR exposure combined with the use of cognitive and relaxation techniques lead to an overall (although non-significant) reduction of the HR and a significant increase of the HRV at the end of the protocol. As the HRV reflects emotional arousal, most notably anxiety, and is known to be a predictor of craving (Newman, Szkodny, Llera, & Przeworski, 2011), the improvement might be due to the reduction of one or both of these factors.

On the technical and creative side, VEs constructed with an inexpensive off-the-shelf graphic engine/GLE were able to generate sufficient presence and craving among the sample of abstinent smokers. In the VRCE group, it is noteworthy that participants mainly selected the beach bar, the workplace and the living room with a balcony for their cue exposure sessions in VR.

Concerning the potential influence of past exposure to electronic entertainment devices, the correlations carried out on the demographic values have revealed that the younger the participants were, the more experience they had with video games, however this experience had almost no effect on perceived presence in VEs. Involvement inside VEs was sought to ensure that participants would behave in a VE as they would when exposed to similar craving cues in reality, the effect of presence is of significant importance. Consequently, correlations between presence scores and self-reported questionnaires or cardiophysiological parameters were calculated and lead to significant and non-significant positive correlations. They seemed to point towards a possible relationship between the degree to which participants felt immersed inside the VEs and the therapeutic outcomes on dependence, craving, mood and cardiophysiology scores as controlled by the autonomic nervous system.

Interestingly, after listing marginal accounts from some of the participants in the VRCE group, we discovered that nausea generated by cybersickness was accidentally associated with tobacco cues as they occurred simultaneously within the VE. Consequently, when exposed to real cigarettes or people smoking outside the clinical settings, these participants reported feeling the same nausea and even disgust. This unscheduled consequence due to punishing conditioning created in VR could be useful in the context of drug aversive therapy. Such aversive procedures are indeed, already exploited for the treatment of alcohol dependence by prescribing antabuse medications. Further investigation of this aspect can be achieved by generating cybersickness voluntarily during VRCE and is a feasible process (i.e. by suddenly lowering the frame rate or provoking external orientational shift of the user's view when seeing tobacco cues).

Even though smoking-related VEs induced craving by sensory stimulations (visual, auditory and proprioceptive) and bodes well for therapeutic efficiency in a clinical setting, we exercise caution in our statements regarding its use in relapse prevention. Several key limitations need to be kept in mind when interpreting these results. First of all, abstinence being one of the inclusion criteria, some participants quit smoking without proper supervision and complained about strong craving and withdrawal symptoms exhibited at the early stage of the protocol. This may explain some of the drop out cases and prompt towards the inception of a more comprehensive care when facing future abstinence. Secondly, this comparative study being the first in smoking relapse prevention, it was decided to isolate the VRCE effect by excluding any other simultaneous form of therapy (most notably NRT or electronic cigarettes). We surmise that the combination of VRCE and other therapeutic instruments could optimize relapse prevention. Future studies should test this assumption for better results. Thirdly, the restricted field of view of the HMD utilized was relatively narrow by today's standard (60° while recent models can cover the complete field of view: 200°). Also, follow-up results are ongoing and will have to demonstrate the VRCE lasting effects. Lastly, the absence of olfactive stimuli such as smoke odours (computer-controlled odour banks can now diffuse specific scents when encountering a targeted event in VR) might have restricted the multisensory integration of the craving cues present in the VEs and limited the craving intensity. *In fine*, we encourage researchers, clinicians and users alike to make the most of the current ubiquitous access of VR, telepsychotherapy, gamification of therapeutic process, recent multi-sensorial equipment with olfactive cues, avatars with a strong artificial intelligence, presence of the therapist inside the VEs themselves and easy access to VE creation for the betterment of mental health in real worlds, metaverses and virtual universes.
